# Clinical Outcome and Cost-Effectiveness of a Synchronous Telehealth Service for Seniors and Nonseniors with Cardiovascular Diseases: Quasi-Experimental Study

**DOI:** 10.2196/jmir.2091

**Published:** 2013-04-24

**Authors:** Ying-Hsien Chen, Yen-Hung Lin, Chi-Sheng Hung, Ching-Chang Huang, Deng-Feng Yeih, Pao-Yu Chuang, Yi-Lwun Ho, Ming-Fong Chen

**Affiliations:** ^1^National Taiwan University HospitalDepartments of Internal MedicineTaipeiTaiwan; ^2^National Taiwan University HospitalDepartments of NursingTaipeiTaiwan; ^3^Graduate Institute of Clinical Medicine, National Taiwan University College of MedicineTaipeiTaiwan

**Keywords:** age factors, telehealth, cost-benefit analysis, cardiovascular diseases

## Abstract

**Background:**

Telehealth based on advanced information technology is an emerging health care strategy for managing chronic diseases. However, the cost-effectiveness and clinical effect of synchronous telehealth services in older patients with cardiovascular diseases has not yet been studied. Since 2009, the Telehealth Center at the National Taiwan University Hospital has provided a range of telehealth services (led by a cardiologist and staffed by cardiovascular nursing specialists) for cardiovascular disease patients including (1) instant transmission of blood pressure, pulse rate, electrocardiography, oximetry, and glucometry for analysis, (2) mutual telephone communication and health promotion, and (3) continuous analytical and decision-making support.

**Objective:**

To evaluate the impact of a synchronous telehealth service on older patients with cardiovascular diseases.

**Methods:**

Between November 2009 and April 2010, patients with cardiovascular disease who received telehealth services at the National Taiwan University Hospital were recruited. We collected data on hospital visits and health expenditures for the 6-month period before and the 6-month period after the opening of the Telehealth Center to assess the clinical impact and cost-effectiveness of telehealth services on cardiovascular patients.

**Results:**

A total of 141 consecutive cardiovascular disease patients were recruited, including 93 aged ≥65 years (senior group) and 48 aged <65 years (nonsenior group). The telehealth intervention significantly reduced the all-cause admission rate per month per person in the nonsenior group (pretelehealth: median 0.09, IQR 0-0.14; posttelehealth: median 0, IQR 0-0; *P*=.002) and the duration (days per month per person) of all-cause hospital stay (pretelehealth: median 0.70, IQR 0-1.96; posttelehealth: median 0, IQR 0-0; *P*<.001) with increased all-cause outpatient visits per month per person (pretelehealth: median 0.77, IQR 0.20-1.64; posttelehealth: mean 1.60, IQR 1.06-2.57; *P*=.002). In the senior group, the telehealth intervention also significantly reduced the all-cause admission rate per month per person (pretelehealth: median 0.10, IQR 0-0.18; posttelehealth: median 0, IQR 0-0; *P*<.001) and the duration (days per month per person) of all-cause hospital stay (pretelehealth: median 0.59, IQR 0-2.24; posttelehealth: median 0, IQR 0-0; *P*<.001) with increased all-cause outpatient visits per month per person (pretelehealth: median 1.40, IQR 0.52-2.63; posttelehealth: median 1.76, IQR 1.12-2.75; *P*=.02). In addition, telehealth intervention reduced the inpatient cost in the nonsenior group from $814.93 (SD 1000.40) to US $217.39 (SD 771.01, *P*=.001) and the total cost per month from US $954.78 (SD 998.70) to US $485.06 (SD 952.47, *P*<.001). In the senior group, the inpatient cost per month was reduced from US $768.27 (SD 1148.20) to US $301.14 (SD 926.92, *P*<.001) and the total cost per month from US $928.20 (SD 1194.11) to US $494.87 (SD 1047.08, *P*<.001).

**Conclusions:**

Synchronous telehealth intervention may reduce costs, decrease all-cause admission rates, and decrease durations of all-cause hospital stays in cardiovascular disease patients, regardless of age.

## Introduction

Elderly individuals are often frail and at high risk for requiring health care. According to Medicare data, 82% of elderly individuals living in the United States have chronic conditions that account for an annual average medical expenditure of US $5015 per person during 1999 [1]. From research based on a representative sample of seniors aged over 65 years nationwide in Taiwan (N=114,873), the prevalence of chronic disease for seniors is 70.4%, and medical expenditures for seniors with chronic conditions accounts for 92.7% of the total medical expenditures for seniors. Among these expenditures, cardiovascular diseases account for an annual average medical expenditure as high as US $4291 per person, which ranks it as the most costly of the chronic diseases in Taiwan [2]. Those patients with multiple comorbidities requiring continued medical care tend to be older and they have often been denied access to hospitals or have faced unequal access to health care, referral, treatment, or other social security benefits in developed countries [3,4].

Long-term chronic health conditions are one of the greatest challenges to worldwide health care systems [5]. Nurse-led case management for disease control and prevention has been successful in diabetes [6], chronic obstructive pulmonary disease [7], coronary heart disease [8], and heart failure [9]. This is particularly true for one of the most deadly diseases in cardiology, chronic heart failure, in which effective management is a major problem because of its high prevalence, high treatment costs, mortality, and morbidity [10]. In addition to heart failure, cardiovascular diseases also include diverse diseases, such as coronary artery disease (CAD), arrhythmia, cardiomyopathy, congenital heart disease, aortic disease, and valvular heart disease. To date, comprehensive home-based heart failure management care systems are thought to be effective in reducing costs and the reliance on prescription drugs, improving clinical outcomes and functional levels, bolstering patient knowledge and self-care, prolonging total survival, enhancing drug adherence, and improving quality of life in the United States, Canada, Europe, and Australia [9,11-13]. Home-based interventions have been shown to improve the clinical outcome and cost-effectiveness of treatment throughout the world despite differences in ethnicity, costs of treatment, differing medical practices, and health care systems [14,15]. The weight of existing evidence led the American College of Cardiology (ACC) Foundation and the American Heart Association (AHA) to recommend multidisciplinary disease management programs, including telemonitoring for patients at high risk for heart failure as a class I indication, with a class A level of evidence [16]. However, 2 prospective randomized multicenter clinical trials, the Telemonitoring to Improve Heart Failure Outcomes (Tele-HF) study in 2010 and the Telemedical Interventional Monitoring in Heart Failure (TIM-HF) study in 2011, used telehealth monitoring for heart failure patients, but did not demonstrate a benefit on mortality rates through telemonitoring [17,18]. The lack of measured benefits was explained as being a product of several factors: inadequate health care professional staffing, information overload, and noncompliance to telemonitoring intervention (in the Tele-HF study). In addition, the TIM-HF trial was unable to detect clinically relevant differences in mortality because of low statistical power.

Advances in modern telecommunication technologies, such as Internet-based rather than telephone-based data exchange platforms, have provided telehealth medicine a new approach for chronic disease management [19]. Synchronous telemedicine involves real-time processing of patient data obtained remotely; therefore, the patient and care provider need to be simultaneously available [20]. However, the effect of synchronous telehealth medicine based on advanced information technology in elderly patients with cardiovascular disease has never been studied. The Telehealth Center at the National Taiwan University Hospital (NTUH) began physician-led synchronous telehealth services in 2009. In this paper, we assess the clinical impact and cost-benefits achieved using telehealth services for patients with cardiovascular diseases.

## Methods

### Patients

The study recruitment strategy followed a nonconcurrent prospective design, approved by the Institutional Review Board at NTUH (Taipei, Taiwan), and conducted by the Taiwan ELEctroHEALTH study group (TELEHEALTH study group). Informed consent was obtained from all participating patients. The TELEHEALTH study group was located in the Telehealth Center of NTUH and launched telehealth services for cardiovascular disease patients in 2009. In order to include those patients with diagnosed cardiovascular disease as well as patients at high risk for cardiovascular disease, patients who had the following disease conditions were considered for enrollment in the telehealth service: (1) CAD with or without percutaneous coronary intervention, (2) myocardial infarction, (3) congestive heart failure, (4) arrhythmia, including bradyarrhythmia, tachyarrhythmia, or ventricular arrhythmia with an implantable cardiac defibrillator implant, (5) diabetes mellitus, (6) syncope, (7) ≥2 CAD risk factors with angina, and (8) other surgical or congenital heart conditions. Syncope, which may be noncardiogenic, was also used as an indication because biometric information obtained through telemonitoring can be of value in cases that are caused from orthostatic hypotension or vasovagal reflex. The main exclusion criterion for the telehealth service was patient refusal. Between November 2009 and May 2010, consecutive cardiovascular disease patients were evaluated for eligibility for telehealth monitoring during inpatient and outpatient treatment. After obtaining informed consent, the patient and main caregiver completed a face-to-face tutorial for operating the manometer, oximeter, glucometer, and electrocardiography device. Internet access and biometrics transmission were confirmed before launching the telehealth service at home. Demographic and clinical data were collected from all participants, including gender, age, occupation, marital status, years of schooling, number of people in the household, main caregiver, clinical comorbidities, biochemistry findings, and echocardiography study results. The estimated glomerular filtration rate (eGFR) was calculated using the simplified Modification of Diet in Renal Disease (MDRD) formula. The clinical expenditure data, including outpatient costs, inpatient costs, emergency department costs, and total cost of all-cause health care and the frequency of health care visits (including emergency department visits, inpatient admissions, inpatient and outpatient procedures, and outpatient physician visits), were collected 6 months before and 6 months after the initiation of the telehealth service.

### Telehealth Service

There were 3 components to the telehealth services provided. First, there were real-time transmission of biometrics from the patients to the health care team, including blood pressure, pulse rate, electrocardiography, oximetry, and glucometry. Glucometry was performed in patients with diabetes mellitus and those with impaired fasting glucose and impaired glucose tolerance. Nondiabetic patients did not have glucometry as a telemonitoring module. Each transmitted clinical biometric was recorded in health record clouds that were under synchronous surveillance by the Telehealth Center at NTUH. Second, there were telephone exchanges between the health care team and patients for communication and health promotion. Third, full-time case managers and cardiologists were in charge of care 24 hours a day. The telehealth services included health education, diet therapy, fluid status evaluation, drug adverse effects evaluation, drug compliance monitoring, mood or emotion care, and patient surveillance through the advanced information technology-based monitor system. Nurse case managers tracked and scrutinized the clinical information carefully and contacted the patient or relatives at least once per day (except in cases in which the patients indicated a preference against daily interactions) or when abnormal data were transmitted back to the service station at the Telehealth Center at NTUH. Patients who were under surveillance by the telehealth service could also contact the service station by telephone when subjective or objective abnormalities were a concern. The clinical information was relayed to the cardiology specialist; the cardiology specialists made the final judgment and suggestions regarding care. The Telehealth Center also provided constant analytical and decision-making support. The management of these heart failure patients was conducted according to the ACC/AHA guidelines for heart failure management [21,22].

### Study Follow-Up and Endpoint

The clinical impact analysis was based on data from outpatient and emergency departments, general wards, and intensive care units. The admission rate, total hospital stay, and hospital costs (including the emergency department, inpatient, and outpatient costs) were collected before and after initiation of the telehealth service. The data were expressed as a monthly average (US $1 equivalent to $31.40 New Taiwan dollars based on the exchange rate on January 14, 2010).

### Statistical Analysis

Enrolled participants were divided according to age into 2 groups: the seniors group included patients aged 65 years and older and the nonseniors group included patients younger than 65 years. Because heart failure is the most-studied cardiovascular disease in the literature and a higher proportion of seniors had heart failure in our study, we further stratified all the participants according to the disease state of heart failure for analysis. Continuous data are presented as mean and standard deviation (SD) and the skewed continuous data are presented as medians and interquartile ranges (IQRs). Discrete data are given as counts (n) and percentages. The Student’s *t* test was applied to compare continuous unpaired data between seniors and nonseniors; for skewed continuous data, the Mann-Whitney test was used. The chi-square test was used to compare categorical data between seniors and nonseniors. The Shapiro-Francia W' test for normality revealed that the changes in clinical outcome were not normally distributed. Therefore, after stratifying the patients according to age and the state of heart failure, the pretelehealth and posttelehealth clinical variables as well as the paired continuous data were compared with the Wilcoxon signed-rank test. A *P* value <.05 was considered statistically significant. Stata/SE 11.0 for Windows (StataCorp LP, College Station, TX, USA) was used for statistical analyses.

## Results

Between November 2009 and May 2010, a total of 141 consecutive cardiovascular disease patients who received telehealth service from the Telehealth Center at NTUH were included in this study. Of these 141 patients, the mean age was 68.5 years (SD 14.4), and the mean duration of telehealth service was 52.7 days (SD 38.2). Among the patients, 56.7% of had preexisting CAD, 18.4% had a myocardial infarction, 39.7% had heart failure, and 44.0% had an arrhythmia. The baseline characteristics are presented in Table 1.

**Table 1:** Baseline demographic data for patients with cardiovascular disease receiving telehealth service.

Demographic data		Nonsenior (<65 years) n=48	Senior (≥65 years) n=93	Total N=141	*P* value
Age (years), median (IQR)		58.0 (49.1-61.0)	76.3 (71.1-80.9)	70.8 (60.8-78.3)	<.001
Female, n (%)		12 (25.0)	43 (46.2)	55 (39.0)	.01
Body weight (kg), mean (SD)		65.3 (15.3)	60.2 (11.6)	61.8 (13.1)	.06
Body height (cm), mean (SD)		165.0 (7.3)	160.0 (8.6)	161.6 (8.5)	.005
BMI(kg/m^2^), mean (SD)		23.7 (4.6)	23.6 (3.9)	23.6 (4.1)	.84
Years of schooling, mean (SD)		13.1 (3.9)	10.4 (4.7)	11.3 (4.6)	.008
Number in household, mean (SD)		2.0 (0.9)	2.2 (1.4)	2.1 (1.3)	.31
Married, n (%)		41 (85.4)	84 (90.3)	125 (88.7)	.39
Telehealth service duration (days), mean (SD)		55.8 (41.8)	51.1 (36.3)	52.7 (38.2)	.49
**Enrollment criteria, n (%)**					
	Coronary artery disease (CAD)	20 (41.7)	44 (47.3)	64 (45.4)	.52
	Myocardial infarction	1 (2.1)	5 (5.4)	6 (4.3)	.36
	Heart failure	8 (16.7)	12 (12.9)	20 (14.2)	.54
	Arrhythmia	10 (20.8)	19 (20.4)	29 (20.6)	.96
	Diabetes mellitus	2 (4.2)	3 (3.2)	5 (3.6)	.78
	Syncope	0	0	0	—
	≥2 CAD risk factors with angina	2 (4.2)	2 (2.2)	4 (2.8)	.49
	Surgical or congenital heart diseases	5 (10.4)	8 (8.6)	13 (9.2)	.72
**Comorbidity, n (%)** ^**a**^					
	Hypertension	27 (56.3)	82 (88.2)	109 (77.3)	<.001
	Diabetes mellitus	14 (29.2)	39 (41.9)	53 (37.6)	.14
	Dyslipidemia	23 (47.9)	36 (38.7)	59 (41.8)	.29
	CAD	25 (52.1)	55 (59.1)	80 (56.7)	.42
	Myocardial infarction	7 (14.6)	19 (20.4)	26 (18.4)	.72
	Heart failure	12 (25.0)	42 (45.2)	56 (39.7)	.02
	Valvular heart disease	4 (8.3)	15 (16.1)	19 (13.5)	.20
	Chronic renal insufficiency	7 (14.6)	34 (36.6)	41 (29.1)	.006
	Stroke	5 (10.4)	22 (23.7)	27 (19.1)	.06
	Arrhythmia	17 (35.4)	45 (48.4)	62 (44.0)	.14
	Atrial fibrillation	8 (16.7)	25 (26.9)	33 (23.4)	.18
	VT or VF	2 (4.2)	2 (2.2)	4 (2.8)	.49
	Bradyarrhythmia	3 (6.3)	14 (15.1)	17 (12.1)	.12
	Pacemaker	6 (12.5)	11 (11.8)	17 (12.1)	.91
**Laboratory results, mean (SD)** ^**b**^					
	LVEF (%)	61.4 (15.5)	58.5 (14.1)	59.4 (14.5)	.18
	Hemoglobin (g/dL)	13.3 (2.8)	12.4 (1.8)	12.7 (2.2)	.04
	Creatinine (mg/dL)	2.0 (2.5)	1.9 (1.7)	1.9 (2.0)	.79
	Fasting glucose (mg/dL)	113.7 (44.9)	111.4 (34.3)	112.2 (38.1)	.76
	Total cholesterol (mg/dL)	186.7 (52.3)	183.9 (44.0)	184.9 (46.7)	.75
	HDL (mg/dL)	41.0 (11.6)	41.7 (13.8)	41.4 (13.1)	.82
	Triglycerides (mg/dL)	142.4 (130.9)	134.6 (87.8)	137.2 (103.9)	.72
	eGFR (mL/min)	64.2 (26.4)	49.7 (22.6)	54.3 (24.7)	.001
**Medical prescriptions, n (%)** ^**c**^					
	Various HTN drugs, mean (SD)	1.6 (1.2)	2.0 (1.0)	19 (1.0)	.06
	Diuretics	14 (29.2)	50 (53.8)	64 (45.4)	.005
	Spironolactone	4 (8.3)	11 (11.8)	15 (10.6)	.52
	Calcium channel blocker	17 (45.4)	40 (43.0)	57 (40.4)	.38
	Antiplatelet	24 (50.0)	54 (58.1)	78 (55.3)	.36
	ACE inhibitor or ARB	17 (35.4)	56 (60.2)	73 (51.8)	.005
	Beta-blockers	25 (52.1)	31 (33.3)	56 (39.7)	.03
	Statin or fibrates	15 (31.3)	24 (25.8)	39 (27.7)	.49
	Antiarrhythmic	15 (31.3)	29 (31.2)	44 (31.2)	.99
	OHA or insulin	5 (10.4)	24 (25.8)	29 (20.6)	.03

^a^ VT: ventricular tachycardia; VF: ventricular fibrillation.

^b^ LVEF: left ventricle ejection fraction; HDL: high-density lipoprotein; eGFR: estimated glomerular filtration rate, calculated using simplified Modification of Diet in Renal Disease (MDRD) formula.

^c^ HTN: hypertension; ACE: angiotensin-converting enzyme; ARB: angiotensin II receptor blocker; OHA: oral hypoglycemic agent.

Patients were further stratified according to age. Seniors (aged ≥65 years) comprised 66.0% (93/141) of the total enrollment with median age 76.3 years (IQR 71.1-80.9 years), and nonseniors (aged <65 years) accounted for 34.0% (48/141) of the total enrollment with median age 58.0 years (IQR 49.1-61.0 years). The indication for enrollment between the senior and nonsenior groups was comparable. Seniors had a significantly higher proportion of females (46.2% vs 25.0%, *P*=.01), fewer years of schooling (mean 10.4, SD 4.7 vs mean 13.1, SD 3.9, *P*=.008), hypertension (88.2% vs 56.3%, *P*<.001), heart failure (45% vs 25%, *P*=.02), and chronic renal insufficiency (36.6% vs 14.6%, *P*=.006). In addition, seniors had a significantly lower level of hemoglobin (mean 12.4 g/dL, SD 1.8 vs mean 13.3 g/dL, SD 2.8, *P*=.04) and eGFR (mean 49.7 mL/min, SD 22.6 vs mean 64.2 mL/min, SD 26.4, *P*=.001). The number of hypertension drugs was comparable between senior and nonseniors; however, compared to the nonseniors, seniors used a significantly higher proportion of diuretics (53.8% vs 29.2%, *P*=.005) and angiotensin-converting enzyme inhibitors or angiotensin II receptor blockers (60.2% vs 35.4%, *P*=.005).

After receiving telehealth services, patients with cardiovascular diseases had a significantly decreased all-cause admission per month rate (pretelehealth: median 0.10, IQR 0-0.17; posttelehealth: median 0, IQR 0-0; *P*<.001), and decreased average days/month duration of all-cause hospital stay (pretelehealth: median 0.60, IQR 0-2.20; posttelehealth: median 0, IQR 0-0; *P*<.001), and an increased all-cause outpatient visits per month rate (pretelehealth: median 1.17, IQR 0.36-2.27; posttelehealth: median 1.70, IQR 1.15-2.72; *P*<.001). There was no significant difference in the all-cause emergency department visit rate.

When stratified according to age, in nonsensiors the average all-cause admission rate per month was significantly decreased (pretelehealth: median 0.09, IQR 0-0.14; posttelehealth: median 0, IQR 0-0; *P*=.002) and the all-cause hospital stay days per month duration was significantly decreased (pretelehealth: median 0.70, IQR 0-1.96; posttelehealth: median 0, IQR 0-0; *P*<.001), whereas the all-cause outpatient visit rate per month was significantly increased (pretelehealth: median 0.77, IQR 0.20-1.64; posttelehealth: median 1.60, IQR 1.06-2.57; *P*=.002). In seniors, the average all-cause admission rate per month was significantly decreased (pretelehealth: median 0.10, IQR 0-0.18; posttelehealth: median 0, IQR 0-0; *P*<.001) and the all-cause hospital stay days per month duration was significantly decreased (pretelehealth: median 0.59, IQR 0-2.24; posttelehealth: median 0, IQR 0-0; *P*<.001), whereas the all-cause outpatient visit rate per month was significantly increased (pretelehealth: median 1.40, IQR 0.52-2.63; posttelehealth: median 1.76, IQR 1.12-2.75; *P*=.02). There was no significant difference in all-cause emergency department visit rates in both sensiors and nonsensiors (Table 2).

**Table 2:** Differences in admission rates and duration of hospital stays 6 months before (pre) and 6 months after (post) initiation of telehealth services in patients with cardiovascular diseases, stratified according to age.

Final measure	Age group, median (IQR)						Total, median (IQR)		
	Nonseniors (<65 years)			Seniors (≥65 years)					
	n=48			n=93			N=141		
	Pre	Post	*P value*	Pre	Post	*P value*	Pre	Post	*P value*
All-cause admission rate^a^	0.09 (0-0.14)	0 (0-0)	.002	0.10 (0-0.18)	0 (0-0)	<.001	0.10 (0-0.17)	0 (0-0)	<.001
Duration of all-cause hospital stay^b^	0.70 (0-1.96)	0 (0-0)	<.001	0.59 (0-2.24)	0 (0-0)	<.001	0.60 (0-2.2)	0 (0-0)	<.001
All-cause outpatient visits^a^	0.77 (0.20-1.64)	1.60 (1.06-2.57)	.002	1.40 (0.53-2.63)	1.76 (1.12-2.75)	.02	1.17 (0.36-2.27)	1.70 (1.15-2.72)	<.001
All-cause emergency department visits^a^	0 (0-0.10)	0 (0-0.20)	.10	0 (0-0.14)	0 (0-0)	.24	0 (0-0.13)	0 (0-0)	.06

^a^ Visits per month per person.

^b^ Day(s) per month per person.

When stratified according to heart failure status, the average all-cause admission rate per month of the non–heart failures was significantly decreased (pretelehealth: median 0.09, IQR 0-0.12; posttelehealth: median 0, IQR 0-0; *P*=.003) and the all-cause hospital stay days per month duration was significantly decreased (pretelehealth: median 0.18, IQR 0-1.03; posttelehealth: median 0, IQR 0-0; *P*<.001), whereas the all-cause outpatient visit rate per month was significantly increased (pretelehealth: median 0.99, IQR 0.33-1.96; posttelehealth: mean 1.48, IQR 0.90-2.40; *P*=.009). The average all-cause admission rate per month of the heart failures was significantly decreased (pretelehealth: median 0.14, IQR 0.10-0.28; posttelehealth: median 0, IQR 0-0; *P*=.005) and the all-cause hospital stay days per month duration was significantly decreased (pretelehealth: median 2.25, IQR 0.97-4.49; posttelehealth: median 0, IQR 0-0; *P*<.001), whereas the all-cause outpatient visit rate per month was significantly increased (pretelehealth: median 1.43, IQR 0.52-2.40; posttelehealth: median 2.20, IQR 1.36-3.53; *P*=.009). There was no significant difference in all-cause emergency department visit rates for heart failure patients and non–heart failure patients (Table 3).

After initiation of the telehealth service, the expenditure during inpatient care decreased per month from US $784.15 (SD 1096.74) to US $272.63 (SD 875.08, *P*<.001), and the expenditure during emergency department care increased per month from US $19.09 (SD 32.82) to US $28.14 (SD 99.82, *P*=.007). Although, the expenditures for outpatient care increased per month from US $134.00 (SD 272.71) to US $190.76 (SD 376.98, *P*=.007), the total cost of all-cause health care decreased per month from US $937.25 (SD 1127.84) to US $491.52 (SD 1012.45, *P*<.001). This pattern of impact remained when patients were stratified according to either age (Table 4) or heart failure status (Table 5).

**Table 3:** Differences in admission rates and duration of hospital stays 6 months before (pre) and 6 months after (post) initiation of telehealth services in patients with cardiovascular diseases, stratified according to heart failure status.

Final measure	Non–heart failure, median (IQR) n=87			Heart failure, median (IQR) n=54		
	Pre	Post	*P* value	Pre	Post	*P* value
All-cause admission rate^a^	0.09 (0-0.12)	0 (0-0)	.003	0.14 (0.10-0.28)	0 (0-0)	.005
Duration of all-cause hospital stay^b^	0.18 (0-1.03)	0 (0-0)	<.001	2.25 (0.97-4.49)	0 (0-0)	<.001
All-cause outpatient visits^a^	0.99 (0.33-1.94)	1.48 (0.90-2.40)	.009	1.43 (0.52-3.40)	2.20 (1.36-3.53)	.009
All-cause emergency department visits^a^	0 (0-0.93)	0 (0-0)	.13	0.10 (0-0.18)	0 (0-0.23)	.38

^a^ Time per month per person.

^b^ Day(s) per month per person.

**Table 4:** Monthly average cost per patient (in US$) 6 months before (pre) and 6 months after (post) initiation of telehealth services in patients with cardiovascular diseases, stratified according to age.

Final measure	Nonsenior, mean (SD) n=48			Senior, mean (SD) n=93			Total participants, mean (SD) N=141		
	Pre	Post	*P value*	Pre	Post	*P value*	Pre	Post	*P value*
Outpatient cost	127.08 (309.34)	263.51 (569.44)	.04	137.57 (253.47)	153.21 (215.45)	.08	134.00 (272.71)	190.76 (376.98)	.007
Inpatient cost	814.93 (1000.40)	217.39 (771.01)	.001	768.27 (1148.20)	301.14 (926.92)	<.001	784.15 (1096.74)	272.63 (875.08)	<.001
Emergency department cost	12.76 (26.89)	4.16 (12.76)	.01	22.35 (35.18)	40.51 (120.93)	.11	19.09 (32.82)	28.14 (99.82)	.007
Total cost of all-cause health care	954.78 (998.70)	485.06 (952.47)	<.001	928.20 (1194.11)	494.87 (1047.08)	<.001	937.25 (1127.84)	491.52 (1012.45)	<.001

**Table 5:** Monthly average cost per patient (in US$) 6 months before (pre) and 6 months after (post) initiation of telehealth services in patients with cardiovascular diseases, stratified according to heart failure status.

Final measure	Non–heart failure, mean (SD) n=87			Heart failure, mean (SD) n=54		
	Pre	Post	*P* value	Pre	Post	*P* value
Outpatient cost	95.47 (213.71)	114.49 (174.57)	.05	196.09 (340.55)	313.64 (548.64)	.08
Inpatient cost	496.94 (748.84)	195.92 (668.84)	<.001	1246.89 (1383.40)	396.22 (1127.68)	<.001
Emergency department cost	12.99 (32.03)	9.52 (32.42)	.06	28.92 (31.94)	58.14 (152.08)	.15
Total cost of all-cause health care	605.39 (777.18)	319.93 (728.71)	<.001	1471.89 (1381.29)	768.00 (1311.42)	<.001

## Discussion

### Principal Results

In our clinical investigation, a real-time telehealth service based on the combination of information technology and rapid-response hospital services improved clinical outcomes, decreased admission rates, and shortened the duration of all-cause hospital stays for cardiovascular disease patients younger than 65 years as well as patients older than 65 years. The telehealth service also provided cost savings by decreasing the expenses of inpatient and total cost of all-cause health care in patients younger than 65 years and in those patients older than 65 years. To the best of our knowledge, this is the first study to demonstrate age differences in the clinical impact and cost-effectiveness of a real-time telehealth service in patients with cardiovascular diseases.

### Comparison to Prior Work

In contrast to traditional case management for chronic diseases, new methods for health promotion and disease control, including Internet-based interventions, telephonic support, home-based interventions, and telemedicine sessions, have been employed for various disease conditions. Telehealth services have been enthusiastically adopted for patients affected by chronic conditions, including chronic obstructive pulmonary disease [23], diabetes self-management [24], renal failure [25], and heart failure [26]. Telemedicine is an efficient approach and is suggested to be an important feature of heart failure management [9,11-13]. Based on advances in telecommunication technologies and their utilization in managing heart failure, 4 generations of telemedicine were proposed: (1) nonreactive data collection and analysis systems, (2) systems with nonimmediate analytical or decision-making structure, (3) remote patient management systems, and (4) fully integrated remote management systems, including invasive and noninvasive medical devices [20]. From the positive telephone support results and ongoing telemonitoring studies, it seems likely that telemedicine will be an efficient approach and become an important feature of heart failure management. However, 2 recent randomized clinical trials in heart failure did not corroborate these findings for morbidity- and mortality-related endpoints [17,18], and another telehealth study including elderly patients with multiple medical issues reported increased mortality rates when telemonitoring was in use [27]. The contradictory results are likely attributed to several factors.

Remote telemedicine delivery methods, such as synchronous (real-time) or asynchronous (store-and-forward) telehealth care delivery [28], vary and this can affect the clinical impact of telehealth care. After receiving the biometrics data through telemonitoring, the manner in which the telehealth service or telemedical remote management professionals utilize the data is one of the most important factors determining clinical impact. In asynchronous telehealth care delivery, the biometric data can be transmitted synchronously or asynchronously. The data are often stored first and reviewed later; therefore, data processing and remote patient management can be delayed, particularly outside of office hours. The Tele-HF study is representative of asynchronous telemonitoring [18] in which biometric data were reviewed by the patient’s physician every weekday; the Tele-HF study showed a negative result of telemonitoring in heart failure patients. In synchronous telehealth care delivery, biometric data are transmitted and processed on a real-time basis. The patient and health care provider need to be simultaneously available, as does the telemedical remote management professional. The Telemonitoring in the Management of Heart Failure (TEMA-HF) [29] and TIMF-HF [30] studies are representative of synchronous telemonitoring which requires a high degree of health professional participation for processing complex incoming physiological data and making subsequent therapeutic decisions. Although the TIM-HF study failed to reveal a clinical benefit, the TEMA-HF trial showed a reduced mortality rate in the telemonitoring group. Variations in the degree of urgency following biometrics transmission may account for the conflicting results of clinical research [20]. For the biometrics transmission and remote patient management to be synchronous, modalities such as telephone touch pad-based telemonitoring [31], video consultation-based telemonitoring [32], and iPhone-based telemonitoring [33] have been used. With advances in modern telecommunication technologies, telehealth has great potential to improve access to remote health care as an adjunct to traditional medical management. However, its adoption in routine health care has been slow, likely due to lack of clarity regarding the value of telehealth implementation [34].

Differences in selected physiological monitoring parameters (eg, body weight, heart rate, blood pressure, body temperature, electrocardiography, oxygen saturation, and fasting glucose) in noninvasive telemedical systems may influence the clinical outcome. However, the question of which physiological parameter provides the best benefit for cardiovascular disease remains unanswered. Alternatively, the solution must be connected to disease severity and the spectrum of disease.

Different diseases of interest or variations in disease severity may result in differences in the efficacy of telehealth services. Heart failure–related diseases with different New York Heart Association functional classes, different stages of chronic kidney disease, and different types of cardiorenal syndrome or associated arrhythmia would fundamentally govern the clinical course.

Social factors, cultural factors, diet habits, social habits, adherence to medical prescriptions, self-empowerment, consumer price index, medical practice behavior, and ethnic differences may govern the results of telehealth service investigations. Indeed, the key to telehealth service success is that the service should offer patients the opportunity to become actively involved in management of their own health care. Information sharing and communication techniques provide the opportunity for health professionals to improve patient health awareness outside of the hospital and ensure that the patient plays an active role in their own therapeutic process. The incorporation of these techniques also will represent a new approach to the therapeutic relationship, which will emulate the traditional face-to-face relationship between health care providers and patients in a remote capacity. Health professionals that adapt these techniques are able to provide consultations related to health promotion, disease prevention, and facilitation of illness recovery in a more responsive manner.

Age is always a major concern in medical practice that should never be overlooked. Older age is usually an independent prognostic factor for infectious disease, acute coronary syndrome, stroke, malignancy, and chronic kidney disease, among others. Despite not actually being considered a minority group, elderly patients may be discriminated against in several health care-related situations. Health insurance providers tend to exclude elderly patients from obtaining comprehensive insurance coverage. In developed countries, issues of restricted access to hospitals or facing unequal access to health care, referral, and treatment remain [3,4]. Clinical trials tend to exclude elderly patients [35]; hence, the conclusions reached in studies of nonelderly populations cannot be extrapolated to elderly patients. As the elderly population continues to grow, geriatrics is a burgeoning science and a subspecialty of internal and family medicine.

There are several concerns about the application of telehealth services for the elderly. First, elderly patients may have more comorbidities and chronic diseases that make periodic inpatient medical care in this group almost inevitable. Therefore, synchronous telehealth service for senior patients may not work as effectively as in nonsenior patients. Second, seniors might not be able to fulfill the telehealth care model completely due to the decline of their physical and mental condition. Utilizing computers and learning new communication technology may be difficult for elderly patients with impaired cognitive and physical function.

However, our study demonstrated that synchronous telehealth service can provide cost-effectiveness benefits as well as clinical benefits for reducing admission rates and duration of hospital stay regardless of age status. We believe that the success was because of the support from caregivers and families who built up the care and fulfilled the complete model of synchronous telehealth service. Although telehealth services cannot prevent diseases or major adverse cardiac events from occurring, the service may still be helpful in early detection of declining health status and assist in the delivery of timely medical therapy. Therefore, cost savings can still be achieved in elderly patients.

### Limitations

There were several limitations of our study. First, the study design lacked randomization, which resulted in the heterogeneity of the patient population and disease severity. There were also no control groups. Therefore, this study was a quasi-experimental study. Second, the relatively short period of follow-up may underestimate or overestimate the cost-effectiveness and clinical outcomes of the telehealth service for patients with cardiovascular disease. Although the initial 6 months of follow-up provided satisfactory results, this initial benefit may not guarantee a long-standing benefit or reflect mortality reduction. Third, the diversity of medical prescriptions and baseline demographic data may potentially confound the results.

### Conclusions

The results of our study suggest that synchronous telehealth intervention may provide cost savings and clinical improvement in cardiovascular disease patients regardless of age status. Particularly in patients aged older than 65 years whose application of telehealth service was in doubt, they also benefit from reduced health care expenditures, reduced admission rates, and decreased duration of all-cause hospital stays when their illnesses are managed by using a telehealth service. In conclusion, telehealth medicine may be an effective model for cost savings in patients with cardiovascular diseases. Due to the limitations of the quasi-experimental study design, these findings require confirmation in a large randomized controlled trial in an older adult population with chronic diseases.

## Abbreviations

ACC: American College of Cardiology
AHA: American Heart Association
CAD: coronary artery disease
eGFR: estimated glomerular filtration rate
MDRD: Modification of Diet in Renal Disease
NTUH: National Taiwan University Hospital
